# Threat of biographical disruption: the gendered construction and experience of infertility following cancer for women and men

**DOI:** 10.1186/s12885-018-4172-5

**Published:** 2018-03-05

**Authors:** Jane M. Ussher, Janette Perz, The Australian Cancer and Fertility Study Team (ACFST), Annie Miller, Pandora Patterson, Gerard Wain, Kim Hobbs, Catherine Mason, Laura Kirsten, Edith Weisberg, Alison Butt, Joanne Cummings, Kathryn Nattress, Chloe Parton

**Affiliations:** Translational Health Research Institute, School of Medicine, Western Sydney University, Locked Bag 1797, Penrith South, 2751 Australia

**Keywords:** Cancer and fertility, Infertility, Parenthood, Gender differences, Gender identity, Adult identity, Biographical disruption, Psychological distress, Health care professional support, Health information

## Abstract

**Background:**

Infertility is a major concern for people with cancer and their partners. There have been calls for further research on the gendered nature of psychosocial, emotional and identity concomitants of fertility post-cancer across women and men.

**Method:**

The gendered construction and experience of infertility following cancer was examined through a survey of 693 women and 185 men, and in-depth one-to-one interviews with a subsample of survey respondents, 61 women and 17 men, purposively selected across cancer types and age groups. Thematic decomposition was used to examine the open ended survey responses and interviews. The chi square test for independence was used to test for group differences between women and men on closed survey items.

**Results:**

In the thematic decomposition, infertility was identified as providing a ‘Threat of Biographical Disruption’ which impacted on life course and identity, for both women and men. Subthemes identified were: ‘Parenthood as central to adulthood’; ‘Infertility as a threat to gender identity’; ‘ Unknown fertility status and delayed parenthood’; ‘Feelings of loss and grief’; ‘Absence of understanding and support’; ‘Benefit finding and renegotiation of identity’. In the closed survey items, the majority of women and men agreed that they had always ‘wanted to be a parent’ and that ‘parenthood was a more important life goal than a satisfying career’. ‘It is hard to feel like a true adult until you have a child’ and impact upon ‘my feelings about myself as a man or a woman’ was reported by both women and men, with significantly more women reporting ‘I feel empty because of fertility issues’. Many participants agreed they ‘could visualise a happy life without a child’ and there is ‘freedom without children’. Significantly more men than women reported that they had not discussed fertility with a health care professional.

**Conclusion:**

The fear of infertility following cancer, or knowledge of compromised fertility, can have negative effects on identity and psychological wellbeing for both women and men, serving to create biographical disruption. Support from family, partners and health care professionals can facilitate renegotiation of identity and coping.

## Background

### Infertility and cancer

Fertility is one of the major concerns confronting people with cancer and their partners [1, 2], with infertility post-cancer being described as a “double trauma” [3], affecting between 25 and 60% of cancer survivors [4]. At the same time, a significant proportion of cancer survivors report a desire for parenthood [5, 6] with fertility conferring feelings of normality [7], and having a child serving to “close the door” on cancer ([8], p.105). However, rates of parenthood among cancer survivors are generally lower than in their non-cancer counterparts [9], particularly for women [10], suggesting parenthood remains an unfulfilled desire for many.

Cancer can affect fertility in a number of ways. Infertility can be caused by the disease itself, or result from gonadal damage secondary to chemo-therapy, radio therapy, or from bone marrow transplantation [4, 11]. This can produce early menopause or uterine damage in women, or retrograde ejaculation and azoospermia in men [11], with survivors of childhood cancer also being at risk of delayed pubertal growth [12]. Secondary infertility following cancer, where parenthood is deferred or avoided, can result from fears that the disease will return following treatment, fear of passing cancer to an unborn child, or anxiety concerning the stress or ability to care for a child [8]. While there have been significant advances in strategies to preserve fertility, these interventions carry their own risks, in terms of ovarian stimulation, surgical risk to ovaries and testicles, as well as risks from delaying commencement of cancer treatment [4, 13]. Fertility preservation treatment failure or suboptimal response can also lead to psychological distress, and loss of hope for future fertility [13].

The consequences of infertility following cancer have been described as “devastating”, resulting in distress, fear and a state of feeling “broken hearted” ([14], p.615), associated with depression, anxiety, grief, lowered quality of life, and low self-esteem [7, 8, 15–18]. Conversely, being fertile is a predictor of good quality of life post-cancer [19]. However, in contrast to the substantial research literature on fertility outside of the context of cancer [20, 21], psychosocial research on fertility post-cancer is an area of relatively recent development, with the major focus of previous research being on treatment, fertility preservation and pregnancy outcomes [5, 11, 22], conducted from a medical perspective [23]. This has led to repeated calls for further research on the psychosocial, emotional and identity concomitants of fertility post-cancer [4, 8, 22, 24].

### The gendered nature of infertility

More specifically, it has been argued that there is a need for psychosocial research examining the gendered experience of fertility concerns following cancer [6]. Fertility is central to gender identity [25, 26], with the psychosocial experience of infertility reported to be different across genders [20, 27]. For men, whilst it may disappointing to be infertile, it has been reported to be ‘not as devastating as impotence or abnormal genitals’ ([27] p155). This stands in contrast to women, for whom biological motherhood stands as a core signifier of adult femininity [27], with infertility positioned as failure to fulfil the role of ‘good wife’ and ‘mother’ [26].

In this vein, a number of studies have reported higher levels of fertility related distress in women cancer survivors [7, 28, 29]. Conversely, reproductive concerns and fears of infertility have been reported across male and female cancer patients [30, 31]. Previous research in this field has been criticised for being small scale, focusing on reproductive cancers, and recently diagnosed young women, with patients recruited from a single clinical site [1, 6]. There is evidence that a wide range of cancers and cancer treatments may impact upon fertility [4, 13], and fertility related distress occurs across tumour type [32]. This suggests a need for inclusion of a broad range of cancer types in research on the experience of fertility concerns following cancer. The primary focus of previous research on adolescents and young adults (AYAs) [1, 6, 33] is reinforced by fertility guidelines which focus on AYAs [34, 35]. This can result in the fertility concerns and experiences of older adults being overlooked [36]: in Australia those over the age of 25 [37], in the USA those over age 39 [38], reflecting different age ranges in the definition of AYA cancer survivor. There is a need for further research to examine the gendered experience of cancer related infertility distress across a broad section of men and women survivors, tumour types, stages and age groups, including patients recruited from a range of contexts.

### Biographical disruption and gender identity

Social constructions of fertility, sexuality and gender identity are influential in determining the ability of individuals and couples to negotiate the psychosocial concomitants of fertility and infertility [20]. A framework of ‘biographical disruption’ [39] has been used to understand the impact of chronic illness, including cancer, on identity and wellbeing. In his seminal paper, Bury [39] described biographical disruption as an experience wherein “the structures of everyday life and the forms of knowledge which underpin them are disrupted” (p. 169). This can affect how people view themselves and how they believe they are viewed by others [40], associated with social isolation and a sense of being different from contemporaries [41], often entailing renegotiation of self and identity [42]. Previous research on biographical disruption following diagnosis of cancer has focused on threat to sexual intimacy and relationships [43–45], psychological distress [46], or experiences of liminality [40, 47]. Whilst there have been suggestions that infertility may be one factor amongst many that serve as a biographical disruption for young people with cancer [48], there has been no previous research that has focused specifically on the potential for biographical disruption following cancer related infertility.

The aim of this study was to examine the gendered construction and experience of cancer related infertility in women and men, across a range of cancer types and age groups, in order to understand the psychosocial, emotional and identity concomitants of compromised fertility, and the potential for biographical disruption.

## Method

### Design

A mixed method design was used, involving a survey completed by a broad cross section of women and men cancer survivors, across cancer types and age groups, and in-depth one-to-one interviews conducted with a purposively selected subsample of survey respondents.

### Eligibility and recruitment

Participants were part of a larger program of research examining the construction and experiences of infertility after a cancer diagnosis, and interactions between patients and health care providers, from the perspective of patients, carers and health care providers [49–51]. Participants responded to advertisements circulated across Australia through social media, media stories in local press, advertisements in cancer and carer-specific newsletters, cancer support groups, hospital clinics, and local Cancer Council Websites and telephone helplines. Inclusion criteria were diagnosis and treatment for cancer, established on a self-report basis. Participants initially completed a survey in an online or postal modality examining their perceptions and experiences of fertility post-cancer [49]. At the end of the survey, participants were invited to volunteer to take part in an interview, to discuss changes to fertility in more depth. Purposive sampling [52] was used to select interview participants, with the aim of gaining insight into the gendered experience of cancer related fertility concerns, across age group, cancer types, and parity. Written consent was provided by all participants.

### Interview and survey

Interviews were conducted one-to-one by telephone, taking approximately 60 min, and were recorded and transcribed verbatim. The topics covered in the interview included: experiences and feelings about parenthood and infertility, the negotiation and experience of fertility concerns within romantic relationships, changes to personal identity and body image since in relation to fertility concerns, and experiences of interacting with health professionals (the latter reported in detail elsewhere) [51]. The interviews were conversational in style, with the wording and formatting of questions used flexibly to suit the particular context of the participant [53]. Professional transcribers transcribed the interviews verbatim, and a member of the research team conducted integrity checking to assess accuracy.

The survey included a series of closed and open ended questions about fertility and cancer. In this paper, we focus on participant responses to a series of items that related to themes identified in the qualitative data. This includes single items selected from the Fertility Problems Inventory (FPI) [54], as well as closed and open ended items devised specifically for this study, examining desire for parenthood, concern about infertility, association between fertility and identity, emotional response to infertility, partner understanding, discussion of fertility with health care professionals, and communication about fertility concerns (see supplementary information).

### Analysis

Thematic decomposition [55, 56] was used to examine the open ended survey responses and interviews. This analytic technique combines discursive approaches with thematic analysis and is informed by the notion that meanings are socially constituted through discourse. The analysis was conducted using an inductive approach, with the development of themes being data driven, rather than based on pre-existing research on fertility and cancer. This process involved all of the team members reading through the open ended survey responses and a selection of interviews in order to identify first order codes such as ‘disclosure of fertility concerns’, ‘benefit finding’, ‘life changes’, ‘negative emotions’, ‘positive emotions and strategies’ and ‘masculinity’. Each team member brought suggestion of the first order codes to the meeting, and the final coding frame was devised through a process of consensus. The entire dataset was then coded by two ACFST team members (KS and JC) using NVivo, a computer package that facilitates organisation of coded qualitative data. The senior member of the team (JU) checked the coding to ensure consistency across codes and coders. All of the coded data was then read through by three members of the team (JU, KS and JC), and summaries of the themes within the coded data produced. Codes were then grouped into higher order discursive themes, focusing on constructions of fertility and infertility, as well as experiences of support and coping. This process involved checking for emerging patterns, variability and consistency, commonality across participants, and for uniqueness within cases, in order to identify the discursive constructions of fertility and infertility and of support, in the context of broader cultural discourse. The function these discourses served for individuals was identified and attention paid to the subject positions made available through various discursive constructions of fertility. Through this process, a final overarching theme was developed from the interviews and open ended survey responses, as well as a number of sub-themes. In the presentation of analysis, pseudonyms were used for quotes from the interviews, with the gender of open ended survey responses indicated by ‘Man’ or ‘Woman’. For longer quotes the current age, cancer type, years since cancer diagnosis, and parenthood status (parous/nulliparous) is provided.

The chi square test for independence was used to test for group differences between women and men on closed survey items. In the presentation of results, we use valid percentages, which reflect the proportion of participants who responded to each item.

## Results

### Participants

Eight hundred and seventy eight people living with cancer (693 women, 185 men) completed the survey. The average age of survey participants was 42.53 years (SD = 14.21), and average time from diagnosis 6.22 years (SD = 7.01). The sample was drawn across cancer types including breast (56.7%), gynaecological (12.9%), hematologic (12.7%), gastrointestinal (4.8%), neurologic (3.2%), head and neck (2.9%), skin (2.3%), musculoskeletal (2.3%), genitourinary (0.9%) and respiratory (0.7%). Disease diagnosis status ranged between early and advanced stages, with 67% reporting that their cancer was diagnosed at an early stage. The sample was almost exclusively heterosexual (98%), with 71% reporting that they were currently in a relationship. Fifty-eight percent of survey respondents reported that they had children, 95% were the biological parent, and 85% had become a parent before cancer. Twenty nine per cent of men (*n* = 49) reported that they had ‘engaged in a fertility preservation investigation or procedure’, compared to 20% (*n* = 130) of women.

Two hundred fifty six people living with cancer (199 women, 57 men) indicated they would be willing to participate in a follow up interview. Of those who accepted the invitation to take part in the interviews, 78 participants aged between 18 and 58 (*M* = 45.10), 61 women and 17 men, were interviewed. We interviewed a larger number of women due to the broader range of experiences reported by women participants, which meant that it took longer to reach saturation, no new information in three successive interviews [57].

### Threat of biographical disruption: Impact of infertility on life course and identity

The final overarching theme developed from the interviews and open ended interviews was: ‘Threat of biographical disruption: Impact of infertility on life course and identity’. There were a number of subthemes, outlined in the thematic map (Table 1), and described in the analysis below.

“It’s is hard to feel like a true adult until you have a child”: Parenthood as central to adulthood.

**Table 1:** Thematic map of analysis

Threat of biographical disruption: Impact of infertility on life course and identity	
“It’s is hard to feel like a true adult until you have a child”: Parenthood as central to adulthood	
*Born to be a parent: Desire for parenthood*	
*Parenthood as central to adult identity:*	
“I feel like an inadequate failure”: Infertility as a threat to gender identity	
* Fraudulent woman: Infertility as a threat to femininity*	
*Inferior and impotent: Threat to masculine identity*	
“The worst part is waiting and worrying”: Unknown fertility status and delayed parenthood	
*Uncertainty and anxiety of fertility status*	
*Difficulty of needing to wait*	
*Impact of cancer on future children*	
*Men avoiding fertility assessment*	
“Mourning the loss of future children”: Feelings of loss and grief	
“Being made to feel more inadequate on a regular basis”: Absence of understanding and support	
*Conflict or withdrawal: absence of partner support*	
*Judgement or discomfort: negative responses of friends and family*	
*Absence of understanding or information from health care professionals.*	
“Having children is second to living”: Benefit finding and renegotiation of identity	
*Accepting a life without children*	
*Nurturing through other means*	
*Re-evaluating life priorities – renegotiating identity*	
*Importance of support from significant others*	
*Health care professional support*	

#### Born to be a parent: Desire for parenthood

Having children was reported to be a long held desire for the majority of participants, with 60% (*n* = 331) of women and 53.8% (*n* = 70) of men who responded agreeing with the survey item that “as long as I could remember I wanted to be a parent” (FPI) (*ns*). This was explained by Deborah (25, Breast, 4 years, nulliparous) who told us: “I’ve always been maternal, and always wanted a baby” and by Ben (20, Leukaemia, 7 years, nulliparous) who said “I’ve always grown up wanting to be a father”. Parenthood was positioned as a taken for granted stage in the normal adult life course that participants assumed they would experience, part of married life: “Even young, I just imagined growing up, getting married, having children” (Jasmine 20, Hematologic, 6 years, nulliparous); “because I was so young I just assumed when I grew older I would get married and have kids like everybody else” (Woman 17, Leukaemia, 10 years, nulliparous). Parenthood was positioned as “a more important goal than a satisfying career” (FPI) by 73% (*n* = 408) of women and 68% (*n* = 94) of men who responded to this survey item (*ns*), demonstrated its relative significance in relation to other life goals.

This desire for children was positioned by many as “innate” or “natural”, with 60.8% (*n* = 331) of women and 53.1% (*n* = 68) of men agreeing with the survey item that they were “born to be a parent” (FPI) (*ns*). Whilst being around other people’s children could provide a “bond”, having your own biological child was described as “special”, with “being able to watch them grow” and the “enjoyment of having a child” valued by many participants, as Laurence, who was a stepfather, told us:

That sort of bond with other boys and sort of being able to be a father figure. I’ve got lots and lots of opportunity to do that, but I guess there’s something special about having your own kids (37 Testicular, 3 years, parous).

The act of being pregnant was positioned as central to the desire for parenthood for some women, as Joanne (31 Breast, 6 years, parous) told us “I’ve always wanted to be a parent and have that growing baby inside”. Tanya (37 Gynaecologic, 3 years, nulliparous) said her “purpose was to bear children” because her body “is very ably built” with “child bearing hips” and “big boobs”. This desire for biological parenthood couldn’t easily be “squashed” in the context of cancer related fertility concerns, as Theresa (32 Gynaecologic, 1 year, nulliparous) told us: “I guess I’ve had this desire to have children in my life… there is just some sort of innate emotion there that, kind of, won’t be squashed [laughter] around having children.”

#### Parenthood as central to adult identity

Having a child was positioned as central to adult identity, with 25% (*n* = 137) of women and 32% (*n* = 42) of men who responded agreeing with survey item that “it is hard to feel like a true adult until you have a child” (FPI) (*ns*). The possibility of infertility following cancer diagnosis and treatment could therefore act as a biographical disruption, which served to “throw our lives out of whack” (Ian 27 Thyroid, 1 year, nulliparous) and threaten adult identity. As Abigail (35 Breast, 2 years, nulliparous) explained: “I don’t know, just saying to me that my identity is suddenly very – very different, even though it wasn’t my true identity. That was really, really hard to – to suddenly have to adjust to”. Before cancer, the prospect of infertility had never been considered by younger participants, meaning that they were unprepared for the interruption of life expectations.

I was never concerned prior to my cancer diagnosis, because I never believed it would ever be a problem in my life. I never anticipated I would one day have cancer, and as a result my fertility would be affected (Woman 19 Hodgkin’s Lymphoma, 5 years, nulliparous).

Knowledge or fear of infertility following cancer could thus be constructed and experienced as an “existential crisis”, due to the potential failure to “leave something of myself on the earth after I die” (Nathan 24 Ewing’s Sarcoma, 1 year, nulliparous). In this vein, a number of women constructed cancer as disrupting their “natural” function: “God made women to reproduce and now I can't. I'll never have a child who resembles me” (Woman 16 Hodgkin’s Lymphoma, 1 year, nulliparous). Life without children was described as “wasted”, “without meaning”, “feeling robbed”, “derailing” and “a missed opportunity” by other participants. It also led to the fear of being “isolated” or “left behind” (Brienne 25 Breast, 1 year, nulliparous), as friends planned or started their families. As Kate (29 Haematologic, 1 year, nulliparous) told us,

It’s a really difficult thing to deal with. And, you know, my age, all of my friends are in that time of life as well and they’ve all having children, they’re on their second and you feel very isolated from, you know, life.

Implicit judgement because of failing to live up to societal expectations of ‘normal’ adult identity, because of cancer, permeated these accounts. As one participant commented: “it seems that in this world you are ‘supposed’ to grow up work get married and have children to live a fulfilled life” (Man 22 Haematologic, 2 year, nulliparous). In combination, these accounts suggest that parenthood is positioned as central to adult identity across genders at both an individual and a social level, with potential impact in both spheres of experience for those who face infertility after cancer.

### “I feel like an inadequate failure”: Infertility as a threat to gender identity

Parenthood is not only central to adult identity, for many individuals it is positioned as central to gender identity. Twenty seven per cent (*n* = 175) of women and 25% (*n* = 43) of men agreed with the survey item that cancer related fertility issues had “affected my feelings about myself as a man or a woman” (*ns*), suggesting a threat to their gender identity.

#### Fraudulent woman: Infertility as a threat to femininity

Many of the women participants equated motherhood with femininity, with infertility questioning the legitimacy of their identity as a “real woman”, “total woman” or a “whole woman”. As Sandra (30 Breast, less than a year, nulliparous) explained: “I felt there was an attack on my femininity, um, my potential to be a mother”. Others reported: “I felt a failure as a woman” (Brienne 28 Lung, 1 year, nulliparous); “It's made me feel fraudulent as a woman” (Woman 58 Breast, 56 years). In each of these accounts, women are comparing themselves to an ideal of femininity aligned with motherhood, against which they are self-positioned as “inadequate”, resulting in “feelings of failure, of loss and never being part of the 'sisterhood'” (Woman 44 Thyroid, 13 years, nulliparous), or “feeling a bit worthless” (Joanne 31 Breast, 6 years, parous). These feelings of inadequacy extended to women’s sense of themselves as an intimate partner or wife, illustrated in the following accounts: “It has been my life goal to have a family and to provide well for them, if I can't do this my belief is that I am a failure as a wife” (Woman 29 Thyroid, 13 years, nulliparous); “I know my husband always wanted to be a father, and I feel guilty for not being able to provide that for him” (Woman 37 Breast, 4 years, nulliparous); and “if I can’t give the man that I love a child, what kind of woman am I?” (Louisa 19 Gynaecologic, 7 years, nulliparous). Other women questioned their legitimacy as a partner in future relationships, and said they didn’t know where they “fit” as a woman, as illustrated in the account below:

I feel like my biological clock is ticking and I am alone…. Not being fertile has contributed to my loss of identity - I have no idea where I fit anymore. I am not a wife, I am not a mother - I have no career. I cry every time I find out someone is pregnant (Woman 42 Breast, 5 years, nulliparous).

For some women, cancer related infertility was associated with the removal of the uterus, ovaries, and breasts, parts of the body that are signifiers of femininity. Loss of these body parts further threatened feminine gender identity: “I’m losing my breasts, ovaries, ability to have children…I guess it removes a lot of your femininity” (Roxanne 25 Breast, 1 year, nulliparous). It could also influence how a woman feels she is seen by her partner, as one woman told us:

Sometimes I think it may be the hysterectomy that affects our relationship more than no children. I feel less of a woman without my uterus and ovaries, and I sometimes think he also sees me the same way. Our intimacy and sex life has been greatly affected by my cancer (37 Breast, 4 years, nulliparous).

Cancer treatment can induce early menopause, resulting in “horrible” embodied changes that were positioned as leaving women feeling “less attractive, less sexual, less feminine” (Gemma 24 Gastrointestinal, 2 years, nulliparous) or “old before my time” (Amy 33 Breast, less than a year, parous), further threatening gender identity. These menopausal changes were connected to, and signified, infertility, as one young woman explained:

The wide hips, the artificial periods and the hormone replacement therapy all seem a bit pointless. I sometimes get angry that my body is probably never going to be able to provide life and sustenance to another being (Woman 16 Neurologic, 3 years, nulliparous).

These accounts suggest that the infertility intersects with other embodied change which women have to negotiate, potentially constituting a double difficulty, if both experiences are constructed as negative.

#### Inferior and impotent: Threat to masculine identity

A number of men equated parenthood with masculinity, positioning “male pride” as “knocked” by the potential threat to fertility after cancer, because “a man is not a man until he can have kids” (Male 41 Neurologic, 11 years, parous). Positioning themselves as inferior in relation to other men, participants reported “being infertile makes me feel inadequate” (Man 22 Musculoskeletal, 8 years, nulliparous), or worried that others would judge them if they were infertile: “others may view you in some negative/pity light (which) makes you feel uncomfortable when questions of children came up” (Man 37 Breast, 1 years, parous). This negative judgement was also feared by men who were single, and who were concerned about rejection if they disclosed infertility in future relationships: “I’ve still got that in my mind that if I do find someone and it gets to that time and I say, ‘Oh, I can’t have kids’, they’re just going to get up and go” (Ben 20 Leukaemia, 7 year, nulliparous); “it’s scary, you could lose a girl, based solely on the fact that you can’t have children” (Nathan 24 Ewings Sarcoma, 1 year, nulliparous).

For many men, fertility and sexual virility were positioned as inherently linked, with the unexpected nature of the loss of both evident in reflections on changes over time, from being young, virile and trying to avoid pregnancy when having sex, to losing virility and fertility following cancer treatment, as Liam explained:

I guess my body just doesn’t work anymore with sex and you’ve spent your whole life to that point thinking otherwise… I see myself as having a lot of sex drive and fertility, you know, naturally, and I’ve never worried about that sort of stuff and all of a sudden, bang. It’s kind of affected how I felt as myself as a man (38 Testicular, 9 years, parous).

Participants positioned themselves as “half a man” (Man 52 Melanoma, 12 years, parous), “not a whole man” (Man 58 prostate, 12 years, parous), or said “I don’t feel complete” (Man 58 Prostate, 12 years, parous) because of the combination of changes to sexual functioning and fertility after cancer treatment. A number of participants also reported that they felt “different” as men because of needing to use the “unnatural means” (Evan 21 Leukemia, 3 years, nulliparous) of assisted reproductive technology to have a child. These men reported feeling “guilty” and “responsible” for “putting their wife through IVF”. As Harry (40 Hodgkin’s Lymphoma, 15 years, parous) told us,

She’s a healthy woman and probably quite capable of conceiving a pregnancy naturally, but she’s had to go through IVF, because of what I had to go through, I mean she’s great, she completely supported me and everything was fine with that, but, you know, it’s a big impact on her.

These accounts suggest men’s gender identity, and their self-positioning as a viable partner or “good husband”, is potentially threatened by cancer related infertility, disrupting their sense of self as a man.

### “The worst part is waiting and worrying”: Unknown fertility status and delayed parenthood

#### Uncertainty and anxiety of fertility status

Before cancer diagnosis, 66% (*n* = 432) of women and 70% (*n* = 121) of men reported no “concern about fertility issues” (*ns*). This reduced to 38% (*n* = 255) of women and 46% (*n* = 80) of men after diagnosis (*ns*). Uncertainty and anxiety was reported to be the primary consequence of fertility status being “unknown” or “unclear”, with participants positioning themselves as “worried”, “scared”, “left in the dark” and “unable to plan for the future”. As Jasmine (20 Haematologic, 6 years, nulliparous) explained “I just don’t like not knowing; not a good feeling”. Other women told us: “I don’t know where my fertility stands at the moment, so I don’t know whether I need to be worried or freaking out that I’m not going to be able to have kids” (Anita 22 Neurologic, 3 years, nulliparous); and “it was a lot about us not panicking until we knew the actual facts” (Lara 23 Haematologic, 4 years, nulliparous). Avoidance of thinking about fertility status was described as one way of dealing with the anxiety, as Imogen (43 Breast, 9 years, nulliparous) told us: “I try not to think about it actually, because if you really thought about it, yeah, it would really, really upset me”. However, this strategy was not always effective, as Imogen continued: “Anxiety about infertility has had the worst impact on my quality of life”.

#### Difficulty of needing to wait

For many participants, attempts to conceive were positioned as “delayed”, “put on hold” (Woman 38 Breast, 2 years, nulliparous) or “put on ice” (Liam 38 Testicular, 9 years, parous) until after cancer treatment was completed, or until the woman was in a stable condition that could support a pregnancy. This “waiting” added to anxiety about future fertility, and future identity as a parent: “It’s really hard, really hard to deal with. I’m not a fan of waiting” (Kate 29 Haematologic, 1 year, nulliparous); “I don’t know whether I would still get my period or if I would be fertile” (Woman 18 Ovarian, 4 years, nulliparous); and “I’m worried about the future” (Spencer 27 Thyroid, 1 year, nulliparous). Some women also reported feelings of anxiety around aging, as they felt that trying to conceive later in life was going to be “harder”. For example, Nina (38 Breast, less than a year, nulliparous) explained attempting to conceive after cancer treatment made her feel “nervous” as she was “getting older”. For others the “need to wait” was associated with a “sense of urgency” (Abigail 35 Breast, 2 years, nulliparous) to conceive as soon as possible after treatment ended. As Sandra (30 Breast, less than a year, nulliparous) informed us, “when you’re young you think you’re invincible and you have all the time in the world and all of a sudden now I’ve got a timeline on there. So I’ve got a sentence on it now, on my fertility”. Conversely, for a number of younger women living with cancer, this sense of urgency had “fast tracked” their plan for having children, leading to “looking at having children earlier (in early to mid-20s) rather than later” (Kate 29 Haematologic, 1 year, nulliparous).

Women participants reported anxiety around the “waiting” for results in relation to fertility preservation, IVF not working on the first attempt, or needing to do IVF multiple times. This was associated with anxiety and uncertainty about only having a “finite number of embryos”, as well as the impact on the body through “pumping hormones into my body every time” (Imogen 43 Breast, 9 years, nulliparous).

#### Impact of cancer on future children

Women also commonly reported feeling worried about the impact of cancer treatments on a future pregnancy and the potential health risks to themselves or the child: “I want to know as much as possible with regards to how it would affect my children and what chance they have of getting cancer” (Woman 19 Non-Hodgkin’s Lymphoma, 4 years, nulliparous); and “I was pretty much told that to risk another pregnancy would not be wise for my health” (Woman 39 Breast, 8 years, parous). A number of women and men participants reported feelings of anxiety and uncertainty about their own mortality in relation to parenting, especially when their prognosis was poor, which in turn affected their feelings about fertility. For example, individuals commented “will I be able to see them [my children] grow up?” (Man 37 Breast, less than a year, nulliparous); “given my prognosis it is probably best that we don’t have another child” (Woman 42 Hematologic, 2 years, parous).

#### Men avoiding fertility assessment

Whilst many women would not know their fertility status until they attempted to conceive, the majority of men could ask for an investigation of their sperm count to determine fertility. However, only a minority of men had undertaken such testing in the years after the cancer treatment, as evidenced by the following accounts: “Don’t know what the effects have been and haven’t been back to find out if I am sterile or not” (Man 21Wilms Tumour, 6 years, nulliparous); “I’m not sure if I’m fertile or not” (Shaun 19 Testicular, 16 years, nulliparous); “I still have no clear definite indicator as to whether I am infertile” (Man 22 Neurologic, 8 years, nulliparous). One explanation for avoidance of fertility testing was that the men did not have “the guts to go in for the test”, as Shaun told us. Another was that “it’s worrying to think that I may never have children as a result of treatment” (Man 18 Neuroblastoma, 17 years, nulliparous), so it was easier to avoid confronting the issue through testing, thus avoiding “worry” about the “devastating” knowledge that infertility was certain.

### “Mourning the loss of future children”: Feelings of loss and grief

The psychological consequences of known or suspected infertility were described as a major challenge by many participants. Significantly more women (37%, *n* = 154) than men (23%, *n* = 24) survey participants who responded agreed “I feel empty because of fertility issues” (FPI) (*X*^2^_(2517)_ = 7.73, *p* = .005). Participants reported feelings of “devastation”, “loss” and “shock” due to the fact that cancer treatment had “taken away their choice” or “threatened” their “parental identity,” meaning that “hopes and dreams” about starting a family were “shattered”. Participants positioned this loss as “upsetting” and “heartbreaking”, associated with “overwhelming sadness” or feeling “gutted”. As Laurence (37 Testicular, 1 year, nulliparous) told us: “I just felt sad all the time. I sort of think that potential losses of you know, being a father and all that goes along with it”. Others shared his sentiments: “I get sad just because it’s a change in what maybe I thought my life would be, and that’s sort of like – that brings up grief of hopes or dreams” (Gemma 24 Gastrointestinal, 2 years, nulliparous); “when you’re actually told that you might not be able to have kids and you actually sit back and think about it, it does hit you with a bit of force…it’s shattering” (Evan 21 Leukaemia, 3 years, nulliparous).

For women who had already had children before cancer, the “loss” was about “missing children” and “incomplete” families, as Charlotte explained: “It’s very sad to have that choice taken away from me and that I’m no longer, something that’s not in my control. I - I can’t choose if I want to have another baby or not” (41 Breast, 4 years, parous). Similarly, Melanie (44 Gastrointestinal, 5 years, parous) told us: “as a mum, to know you can’t have that child that you wanted is still really, really hard, hard to deal with”. This sadness could extend to wider family members, demonstrating the far reach of cancer related infertility:

The glaringly obvious thing that’s missing from our lives is more children that we wanted to have. The situation has caused a lot of sadness in my family, because I’m the only child that’s married, and we only have one son and he’s the only grandchild. My parents and my brother and sister, it’s been a very, very sad thing for them as well (Fiona 36 Breast, 2 years, parous).

Many of the women participants characterised living through the “sense of loss and void” (Woman 51 Breast, 13 years, nulliparous) as a “grieving process”, with cancer related infertility resulting in “mourning the loss of future children” (Victoria 49 Gynaecologic, 13 years, parous). This was because “that whole image of the potential family that you had in your head was gone” (Sandra 30 Breast, less than a year, nulliparous). This grieving was described as a feeling of having “lost out on something profound” (Georgina 43 Breast, 10 years, nulliparous), or an “open wound” resulting in “the most raw pain I have ever known” (Woman 45 Breast, 6 years, nulliparous), demonstrating the magnitude of the feelings. As Kate explained:

[Crying]. It’s a loss. It’s an emptiness and a loss. Like, you know, when someone passes away, you feel like that. And you fought so hard to survive and to get through the treatment but, you know, you might never get to have that family that you always wanted. That’s how you feel (29 Haematologic, 1 year, nulliparous).

In a similar vein, Genevieve (44 Gynaecologic, 1 year, nulliparous) stated “your children are born in your heart before they’re ever born in your womb…I never got the chance to say goodbye to the dream of those children.” A number of women participants positioned the impact of infertility as more distressing than the experience of cancer, as Jemima (43 Gynaecologic, 9 years, nulliparous) told us:

I was just devastated. And people thought that I was upset about the cancer, but it wasn’t. It was the fact I could never have kids. And even now, what, nine years later, it still upsets me.

Others talked about the knowledge of infertility compounding cancer related distress: “it felt like you were hit by a brick and then another one” (Woman 37 Breast, 3 years, nulliparous). These accounts demonstrate both the magnitude, and the long lasting impact, of cancer related infertility.

### “Being made to feel more inadequate on a regular basis”: Absence of understanding and support

#### Conflict or withdrawal: Absence of partner support

Support and positive communication was central to participants’ experience of meaning making and in the context of fertility concerns after cancer, as well as central to experiences of information and support seeking. Lack of understanding, or a negative response, on the part of partners, was thus a potential source of distress. Of the survey respondents 43% (*n* = 99) of women and 37% (*n* = 18) of men (*ns*) who responded agreed that “my partner does not understand the way fertility issues affect me” (FPI). This lack of understanding was associated with reports of “conflict” in relationships, or the participant feeling “let down”, evidenced in the following accounts: “I really felt like he let me down. We used to argue about it, and he just said to me once, ‘When are you going to stop saying this to me?’” (Abigail 33 Breast, 2 years, nulliparous). Partners were positioned as “angry”, “disappointed”, “depressed”, “withdrawing”, “upset” and “rejecting” by both men and women participants, as a result of facing the future in a relationship without children. In a number of cases this led to relationship breakdown, as illustrated in the following accounts: “it’s finished our marriage pretty much, my experience with the cancer and infertility” (Charlotte 43 Breast, 4 years, parous); “my wife divorced me because she said that she had found someone who could make her a whole woman” (Man 63 Prostate, 31 years, parous).

#### Judgement or discomfort: Negative responses of friends and family

The reactions of friends and family, in particular their questioning of a woman’s fertility status or options, were also described as compounding fertility related distress and biographical disruption, making it “harder”, because “no-one knows what to say” (Female 37 Breast, 2 years, parous), or asks “the wrong questions”, as evidenced in the following account:

People should be aware of the five stages of mourning. When you receive the bad news about fertility issues you have to go through those five stages to reach acceptance of your reality. If our friends/family interrupt that journey by asking the wrong questions (e.g. what are your options? what are you going to do? etc) it makes dealing with the whole issue a lot harder (Woman 39 Hodgkin’s Lymphoma, 12 years, nulliparous).

The assumption that others have a “right” to “ask and make a judgement” about a woman’s fertility status after a cancer diagnosis was described by one participant as “unhelpful to general wellbeing” and “a constant reminder of what I've been through” leading to “being made to feel more inadequate on a regular basis” (Woman 31 Gynaecological, 1 year, nulliparous). This lack of understanding and inappropriate “comment” about fertility, particularly by people who have children, was described as a “torture” by one woman:

I lost my albeit shaky relationship, my self-worth, my dignity and it would appear my hope - so many hopes and hopes of a normal family. Is that really such a big ask, a normal family? Those that have children already will never understand the comment and torture they put those who cannot have children through (Woman 49 Breast, 2 years, nulliparous).

In a similar vein, when men disclosed their fertility status, or their uncertainty, to friends and family the reactions were sometimes described as “awkward”, “embarrassed”, or gaining “weird looks”. Anticipation of such reactions resulted in an absence of discussion, as Kevin (27 Sarcoma, 5 years, nulliparous) told us: “Friends that are close enough don’t feel comfortable talking to us. So I haven't really talked to anyone about it”. If discussion did take place, it could consisting of the dismissive comment “you can still adopt”, or did not help men to “resolve the issue” (24 Musculoskeletal, 1 year, nulliparous), as there “was more explaining than discussing” (21 Haematologic, 6 years, nulliparous). Absence of empathy or understanding was also evident in some accounts of inappropriate comments, such as “jokes about having only one ball” (Christian 37 Testicular, 10 year, parous). While this was a way for men to talk about fertility using humour, it was upsetting to the man diagnosed with cancer, experienced as emasculating.

#### Absence of understanding or information from health care professionals

A significantly greater proportion of men (64%, *n* = 94) than women (43%, *n* = 280) (X^2^_(2517)_ = 6.54, *p* = .011) reported that they had not discussed fertility with a health care professional since diagnosis of cancer. Lack of “understanding” or absence of “sympathy” when fertility concerns were raised with health care professionals were positioned as a further source of “sadness”, “upset”, “distress” and “dissatisfaction”. This lack of understanding is evident in the following accounts: “sometimes the doctors seemed less than compassionate. Being robbed of your fertility at the age of 25 is rough” (Woman 25 Ovarian, 1 year, nulliparous); and “I was shocked. I’m only 19!!!!!! No-one cared or supported me… I was shocked and hurt, unsupported and alone” (19 Male Leukaemia, 2 years, nulliparous). Having children already, fertility being a “taboo” topic to discuss with the young, or the notion that they should simply be “grateful to be alive” after cancer, were the primary reasons individuals felt their fertility was “dismissed” when raised, or was seen as “something that wasn’t valued, wasn’t important” (Tanya 37 Gynaecological, 3 years, nulliparous). For example, participants told us: “one of the doctors said ‘you’ve had one, be happy with that, don’t be greedy’” (Woman 40 Ovarian, 5 years, parous); “the impression I got was that I should be content to be alive. I have no right to expect to also be fertile” (Woman 43 Breast, 10 years, nulliparous);

Because I was age 16 and in a Children’s hospital when I was diagnosed the discussion on fertility was never brought up because it was seen as a taboo that cannot be spoken about, because it isn’t an issue at the time (Woman 19 Leukaemia, 3 years, nulliparous).

In other cases, participants positioned distress as due to not being informed about the consequences of cancer treatment on their fertility, only finding out themselves when it was “too late”: “I was very dissatisfied that the oncologist did not mention that I may or may not be able to have more children after my radiation treatment. I was only in my thirties at the time” (Man 41 Brain Tumour, 11 years, parous); and “It was very shocking how I found out through a random conversation with friends and not have my doctor tell me or my mum” (Woman 17 Sarcoma, 10 years, nulliparous). Other participants *were* provided with information, but positioned the delivery as “blunt”, “blatant”, “confusing”, “inaccurate”, “uncaring”, “rushed”, or provided “briefly in passing” and “not followed up”. For example, one woman only realised she was infertile following cancer treatment when her doctor commented “infertility is not an excuse for unprotected sex” (Jasmine 20 Haematologic, 6 years, nulliparous). Another found out when her doctor justified a full hysterectomy with the statement “well you can’t get pregnant anyway, so why bother keeping the uterus” (Woman 42 Liposarcoma, 10 years, nulliparous).

Absence of information about “the options that were available” (Woman 38 Breast, nulliparous) for women’s fertility preservation was positioned as a further source of distress. Many women described feeling “regretful” and “sad” that they hadn’t been told, and it was now too late: “If I had have been told upon diagnosis that I may require chemotherapy I could have had some eggs frozen but I wasn't informed until the last minute as chemotherapy wasn't standard treatment with my cancer” (Woman 25 Head and Neck, 1 year, nulliparous). A number of men reported distress because of not being informed about sperm donation prior to radiation therapy: “I honestly didn't know and I wish that I had, because it would just know what it could do it us, even freezing my sperm could have saved us” (Man 41 Brain Tumour, 11 years, parous). Others were provided with information, but reported “confusion” about the reason behind sperm collection: “as an 18 year old, I found the process confusing, rushed, bewildering and confronting” (Man 18 Lympohoma, 1 year, nulliparous). Distress was also associated with discussion of fertility and sperm collection, particularly if it took place in front of parents: “being young, I was furious. Having to give a sample and talking about it was humiliating” (Man 21 Osteosarcoma, 8 years, nulliparous). This account concurs with the positioning of sperm collection as “awkward and daunting”, “awful”, “nerve wracking”, and “embarrassing” from the majority of adolescent and adult men.

### “Having children is second to living”: Benefit finding and renegotiation of identity

#### Accepting a life without children

Challenges to gender identity and feelings of distress were not an inevitable long term consequence of cancer related infertility.

A number of participants acknowledged that they could be happy without children, or that there could be benefits to a life without children, as a counterpoint to experiences of loss and grief. In this vein, 62% (*n* = 335) women and 55 (*n* = 71) men (*ns*) who responded agreed with the survey item ‘I could visualise a happy life without a child (or another child)’ (FPI). This was reflected in open ended responses, as one participant commented “I learnt that you can live a fulfilled life without children” (Man 22 Lymphoma, 2 years, nulliparous). Others described benefits of not being a parent, with 62% (*n* = 308) women and 57 (*n* = 72) men (*ns*) who responded to this survey item agreeing ‘there is a certain freedom without children that appeals to me’. Others commented: “I do joke that I love to travel and I’m spending my children I don’t have’s school fees” (Georgina 43 Breast, 10 years, nulliparous). Having more time for “thinking more about my career” (Francesca 29 Breast, 4 years, nulliparous), and for “focusing on our relationship…doing whatever we want” (Polly 33 Breast, 5 years, nulliparous) was also commonly reported.

#### Nurturing through other means

Accepting a life without children did not exclude experiences of nurturing, as Heather (49 Gynacologic, 25 years, parous) commented: “there’s lots and lots of ways you can give your love out to children in the world, and it doesn’t have to be through your own procreation”. Laurence (37 Testicular, 1 year, nulliparous) described how he and his male partner “sort of looked at the positives of just being us and uncles and friends of people with kids”. Duncan (47 Testicular, 1 year, nulliparous) dealt with the “deep down” feeling that “I would have loved to have had my own child” by focusing on his relationship with his “two young nieces”, reflecting that “when they come and visit and they leave, I think, ‘oh thank God they’re gone’ [laughs]”. Parenting through adoption or fostering was another option as “there are so many children that do need a safe and secure place to live” (Amy 33 Breast, less than a year, parous). Helping friends with their children, starting a playgroup for friends’ children or through church group, or sponsoring third world children in need, were other means of fulfilling the desire to nurture. Other channelled their nurturing into caring for cats or dogs, describing “mothering them like they are children” (Abigail 33 Breast, 2 years, nulliparous). Participants who already had children attempted to reconcile their feelings of disruption of life expectations with the knowledge that they had at least had one child: “it’s had an impact, only having one child rather than two, but one is better than nothing” (Nina 38 Breast, less than a year, parous).

#### Re-evaluating life priorities – Renegotiating identity

A number of participants provided accounts of re-evaluating life’s priorities and finding meaning or benefit in the experience of cancer and infertility. Identity was renegotiated or ‘reconstructed’ [58] as a result. A number of participants positioned themselves as focused on “enjoying just being alive” (Nathan, 24, Ewings Sarcoma, 1 year, nulliparous), or feeling “very grateful for [laughs] what I have now, for being alive [laughs]” (Miranda 38 Gynaecologic, 5 years, parous). This resulted in feeling “pretty lucky” because “I could have been damaged a lot worse than I came out with”, as Eleanor (19 Haematologic, 18 years, nulliparous) told us. “Having children” was positioned as “second to living” (Man 22 Leukaemia, 2 years, nulliparous) in some accounts, with health prioritised over fertility, evidenced in the following accounts “I'd rather have the best shot of living than not being fertile” (Man 21 Lymphoma, 1 year, nulliparous); “being healthy is more important to me” (Woman 33 Breast, 2 years, nulliparous).

The centrality of fertility to gender identity was challenged by a few participants, who told us: “I am a woman by genetics and actions and looks not because I can have a child, or have breasts, or have ovaries, or have children” (Woman 40 Breast, 2 years, nulliparous); “I have never felt that I wasn't a "real" woman due to not having children” (Woman 43 Gynaecological, 2 years, nulliparous); “I’d say I haven’t been emasculated or it doesn’t make me feel any less of a man” (Nathan 24 Ewing’s Sarcoma, 1 year, nulliparous); “there is more – much more to being a man than having children” (Greg 26 Hodgkin’s Lymphoma, 10 years, parous). The judgment of others in this sphere was dealt with by defiance in a number of accounts, illustrated by Georgina’s comments below:

I find it very difficult to see that being a mother fulfils my womanhood or anything. So that’s more about how other people see me than how I see myself. I make the joke that ‘I’m a barren woman’, and I suppose I do that to confront other people with it (43 Breast, 10 years, nulliparous).

#### Importance of support from significant others

Support from partners, family and friends were positioned as a key factor in the development of strategies of adaptation and coping, and a buffer for feelings of inadequacy or distress. Many of the participants reported that sharing their fertility concerns with their “very supportive partner who I can talk to about these sorts of things” (Amy 33 Breast, less than a year, parous) created a feeling of “doing this together” (Man 33 testicular 35, nulliparous), which “made our relationship even stronger” (Evan 21 Hematologic, 3 years, nulliparous), or “opened up a lot deeper dialogue of communication between us” (Sandra 30 Breast, less than a year, nulliparous). This was reflected in the 86% (*n* = 182) of women and 65% (*n* = 30) men (*X*^2^_(2517)_ = 11.57, *p* = .002) who responded to this question agreeing that ‘my partner and I work well handling questions about our infertility’ (FPI). These supportive relationships were positioned as serving to counterbalance feelings of isolation and a sense of being “different” or “inferior”, thus facilitating identity renegotiation. As one survey respondent explained: “My wife was very supportive. I felt like she understood that we might not be able to have more children and she fully supported me and never made me feel inferior or that I let her down” (Man 42 Testicular, 5 years, parous). Others talked of anxiety diminishing through partner support: “I usually just get a very long hug and by the time that’s over with I’ve calmed myself down” (Brienne 28 Breast, 1 year, nulliparous); “he is amazing and while I get little irrational about it all he says its ok and we then focus on us” (Woman 43 Breast, 7 years, nulliparous). Friends and family members could also provide necessary support, allowing participants to “feel that I’m not alone”, “stay positive” or “look to the future”.

#### Health care professional support

Of those who reported discussing fertility with a health care professional, 65% (*n* = 242) of women and 69% (*n* = 54) (*ns)* of men were satisfied with the discussion. Participants who felt supported by health care professionals in relation to fertility concerns, who received information about the impact of treatment on fertility, or who were able to engage in fertility preservation, reported feeling “satisfied”, “positive,” “accepting” and “heard” in relation to their fertility concerns, and were more likely to report identity reconstruction. Support from health professionals made them feel that their fertility concerns were “understood”, and as a result that they were “normal” and “not alone”: “speaking to my psychologist was great, he really understood my issues, helped me understand a lot of the things going on in my head, fears and what not” (Man 24 Ewings Sarcoma, 1 year, nulliparous). Being treated with “respect” and the provision of “accurate” and “detailed” information in order to inform fertility decision making was highly valued, serving to alleviate distress: “a book given to me from CanTeen (youth cancer service) provided great information and helped a lot” (Man 18 Lymphoma, 7 years, nulliparous); “I felt that the doctors treated me with respect and gave me all the information I needed to make an informed decision. They gave me options” (Woman 29 Breast, less than a year, nulliparous).

Facilitation of fertility preservation was also a source of “reassurance” and “hope” that resulted in individuals feeling “cared for”: “My Dr did all the research and recommendations to IVF. He even came out of surgery to call me about his findings. That is what I call a Dr who cares” (Woman 24 Endometrial, 1 year, nulliparous); “it was just so important - I simply cannot explain how much it meant. I am forever grateful to my oncologist” (Woman 42 Breast, 5 years, nulliparous). Equally, a number of men reported that sperm banking helped eased their worries about future infertility as they had a “backup plan” which could act to “cover our bases.”

## Discussion

The findings of this study confirm previous reports that compromised fertility is a significant concern for both women and men diagnosed and treated for cancer, which can negatively affect identity, well-being and life planning [5, 7, 18, 27, 59]. Compromised fertility is rarely anticipated [60], and fear or knowledge of cancer related infertility can therefore serve as a biographical disruption [39], challenging expectations of a normal life course and identity [26]. In the present study, this was associated with a sense of normal life course being disrupted, and feeling different from contemporaries, as has been reported in previous research on cancer and biographical disruption [40, 41]. The findings of the present study suggest that infertility can be a key factor in this experience of cancer related identity dislocation and disruption, not only for young people, as reported previously [48], but also for older adults. This reinforces the need for cancer patients of a reproductive age to have access to psychological support for fertility related distress and biographical disruption, both at the time of diagnosis and into survivorship [61, 62].

Whilst previous research has suggested that parenthood is of greater importance for women than for men [20], we did not find any significant differences across genders, with the majority of women and men participants positioning parenthood as something that they had always wanted, as an innate or natural experience, and as a more important life goal than a satisfying career. This is not surprising, as the majority of individuals expect to become parents [60], with adolescents and young adults conceptualising parenthood as a central component of being “grown up” [63]. These expectations are located in constructions of normality within a ‘pro-natalist’ cultural context [20], where biological reproduction and child-rearing is socially valued and supported [64]. This provides insight into why the threat of infertility can have negative psychological consequences for both women and men affected by cancer.

Fertility is central to the construction of gender identity, and to the gendered performance of women [26, 65] and men [25, 66], with infertility associated with feelings of inadequacy and failure across genders [22, 67], as we found in the present study. This suggests that the impact of impaired fertility on survivors’ intrinsic sense of self as a woman or a man needs to be acknowledged by clinicians working in cancer care. The meaning of infertility has been reported to be fundamentally different across genders [20, 26, 27], as evident across a number of themes identified in the present study. It has been argued that men are concerned about loss of control and their partner’s reaction to infertility [68], whereas women experience infertility as a direct blow to their self-identity [69]. This suggests that information and support services, as well as decision making aids, need to take into account the gendered nature of cancer related infertility distress. The centrality of motherhood to idealised constructions of femininity [70] may explain the significantly greater reports of emptiness, as well as reports of loss and grief, associated with infertility in the present study, as well as higher rates of distress reported by women in previous research [7, 28, 29, 60]. This was reflected in our analysis of the primary outcome measures [49], where women reported significantly higher infertility-related distress than men. This suggests that women may have a greater need for psychological support in the context of cancer related infertility. For men, both heterosexual and gay/bisexual, sexual difficulties after cancer have been reported to act as a greater source of distress than infertility [71–73], confirmed by the findings of the present study. This suggests that health care providers need to be aware of the association between sexuality and infertility, particularly when talking to men. It has previously been argued that psychological interventions for infertile couples should take gender-specific aspects into account, and that more research is needed to address the gender-specific aspects of psychological interventions for infertility [74]. The same could be said for psychological interventions to address infertility in the context of cancer.

Threat to identity resulting from cancer related infertility has been reported to be more common in individuals who identity as heterosexual [75], who formed the majority of participants in our study, as masculinity and femininity are performed through engagement in hetero-normative gendered practice [76]. However, lesbian, gay and bisexual identified participants also reported fertility concerns, and infertility is an issue for many lesbian, gay, bisexual, transgender and intersex (LGBTI) individuals [77]. There is currently an absence of research on LGBTI fertility concerns in the context of cancer, as well as an absence of targeted information and support. Recently, the American Society of Clinical Oncology [78] concluded there is “insufficient knowledge about the health care needs, outcomes, lived experiences and effective interventions to improve outcomes” for LGBTI populations. As a result, health care providers and policy makers are ill-equipped to provide culturally-competent advice or assistance to LGBTI cancer survivors and their families [78, 79].This reinforces the conclusion that attention needs to be paid to the intersection of gender and sexual orientation in the experience of fertility concerns after cancer.

Accounts of identity negotiation and reconstruction [40, 58] illustrate the fluid nature of identity and the ways in which a ‘new normal’ [47] can be develop after diagnosis and treatment for cancer. It also illustrates the ways in which individuals can cope with the consequences of cancer [80, 81], and find benefit in the cancer experience [82, 83], extending previous research to demonstrate coping and benefit finding associated with cancer related infertility. The experience of identity reconstruction and positive coping was not universal across participants, confirming previous reports that benefit finding and positive coping is not experienced by all individuals with cancer, or at all stages of the disease trajectory [81]. Many women, in particular, gave accounts of continuing grief and feelings of failure associated with infertility, with little sense of optimism for a future without children, often many years after diagnosis. This confirms reports that infertility distress can be the most difficult long term effect of cancer, particularly for women [7, 8, 16, 17]. Our finding that such experiences were found across cancer types and age groups suggests that the long term negative consequences of infertility for women need to be acknowledged by policy makers and clinicians, in the context of the gendered meaning and experience of infertility. It would be worthwhile to further explore how this distress persists post reproductive age, ideally using a longitudinal design, in order to inform targeted support and information at different stages of cancer survivorship.

The qualitative analysis presented in the present study provides insight into previous findings that infertility related distress is higher in cancer patients who do not have children [59, 61], findings also reflected in our analysis of primary outcomes, where nulliparous status was significantly associated with infertility-related distress for both women and men [49]. However, a number of women and men who were parents before cancer also gave accounts of biographical disruption, suggesting that the fertility needs and concerns of this group of cancer survivors also needs to be acknowledged, and fertility concerns taken seriously, even if cancer patients have had children prior to diagnosis.

Support from a partner, or from family and friends, can alleviate infertility related distress after cancer [2], and increase the likelihood of identity reconstruction and coping [80], as was reported in the present study. This supports the contention that cancer is a ‘we-disease’ [84], and that the perspectives of partners and other carers need to be considered in understanding the psycho-social impact of infertility following cancer [85]. However, infertility can result in strain within intimate relationships [86, 87], and some individuals are reluctant to disclose their fertility status because of perceived social stigma [22, 88]. This may influence identity reconstruction and coping with compromised fertility after cancer. These findings reinforce the importance of including partners in information provision and supportive care associated with fertility concerns following cancer treatment. Further research is needed to explore the impact of cancer related infertility on partners and other family members [61], and the ways in which partners can facilitate patient coping.

Our findings support previous reports of the positive role of health care professional support in facilitating benefit finding and coping after cancer [80], as well as reports that levels of fertility related distress are lower in individuals who have received pre-treatment information about the impact of cancer on fertility [89], counselling about options for fertility preservation [90], and who are satisfied with the information provided [4, 91]. However, there is evidence that many health professionals do not engage in discussions about fertility after cancer [29], and that such discussions when they do take place are not always satisfactory for patients and their carers [51], which may have long term consequences for patient well-being and coping. The findings of this study reinforce the viewpoint that discussion of fertility concerns by clinicians is “a crucial aspect of high quality healthcare” which helps with patient adjustment [92], and with the threat of biographical disruption. There is also a “duty of care” to provide information about options for fertility preservation, and referral to a fertility specialist, to protect against the long term impact of interrupted childbearing and allow patients and their partners to form a biological family after cancer treatment [61]. As fertility preservation is not always successful, and can be associated with uncertainty and anxiety [62], as reported in the present study, the use of decision aids [93] to facilitate patients in their choice of whether or not to engage in fertility preservation is a much needed recent development, which needs to be made widely to patients of reproductive age. Fears that the health of future offspring may be impacted by cancer history suggests that fertility counselling and decision making support should include not only reproductive function, but the thoughts and feelings patients have surrounding their fertility potential and the impact this has for their quality of life throughout the cancer journey [61, 94]. A recent review of studies evaluating psychological and educational interventions to address infertility outside of the context of cancer concluded that such studies were generally poorly designed and executed [95]. There is also a need for studies employing appropriate methodological techniques to investigate the benefits of psychological interventions to address fertility concerns and infertility after cancer.

This study had a number of strengths and limitations. The strengths were the use of a survey of large sample of men and women, across cancer types and age groups, and qualitative interviews to examine subjective accounts of infertility in depth from a gendered perspective. Further research is needed to explore how gendered-based experiences interact with other key factors in order to have a clear picture of who may be most at risk for experiencing distress and how to address such issues clinically and with a targeted approach. The focus on psycho-social concomitants of infertility, the social construction of gendered experiences, and the use of a framework of biographical disruption, is also a strength addressing gaps in the previous research. The limitations include the fact that participants were recruited as part of research study examining experiences of fertility after cancer, which may have resulted in a greater focus on infertility within the accounts. The cross sectional nature of the data is also a limitation. Future research using a longitudinal design could usefully examine biographical disruption and reconstruction of identity at different stages of the cancer journey. As fertility preservation can influence adaption to the threat of infertility following cancer [61], further research is needed to examine the subjective experience of fertility concerns in those who have, and have not, undergone such procedures.

## Conclusion

In conclusion, this study illustrates that the fear of infertility following cancer, or knowledge of compromised fertility, can have negative effects on identity and psychological wellbeing for both women and men, serving to create biographical disruption. Many individuals were able to develop coping strategies that facilitated identity reconstruction, facilitated by support from family, friends and health care professionals. None of these strategies were positioned as compensation for infertility. However, they demonstrate that information and support, and the capacity to re-evaluate life and identity, as well as the ability to find alternative sources of fulfilment and nurturing, can serve to reduce distress and the negative impact of biographical disruption associated with cancer related infertility. Fertility concerns need to be addressed by clinicians working with individuals of reproductive age who are diagnosed and treated for cancer, in order to alleviate distress and facilitate identity renegotiation in the face of biographical disruption. The opening of dialogue with patients to discuss their fertility concerns and needs within oncofertility will allow patients to be better supported emotionally, minimise long term distress, and facilitate referral to specialist fertility services to gain additional support.

## Abbreviation

FPI: Fertility problem inventory
